# Phytochemicals as Modulators of Paraoxonase-1 in Health and Diseases

**DOI:** 10.3390/antiox11071273

**Published:** 2022-06-27

**Authors:** Zahra Najafi Arab, Danial Khayatan, Seyed Mehrad Razavi, Kimia Zare, Elnaz Kheradkhah, Saeideh Momtaz, Gianna Ferretti, Tiziana Bacchetti, Thozhukat Sathyapalan, Seyed Ahmad Emami, Amir Hossein Abdolghaffari, Amirhossein Sahebkar

**Affiliations:** 1 Department of Toxicology & Pharmacology, Faculty of Pharmacy, Tehran Medical Sciences, Islamic Azad University, Tehran, Iran; zahraa.naajafi@gmail.com (Z.N.A.); danialkhayatan@gmail.com (D.K.); mehradrazavi1376@gmail.com (S.M.R.); elnazkheradkhah1997@gmail.com (E.K.); 2 School of Medicine, Shahid Sadoughi University of Medical Sciences and Health Services, Yazd, Iran; kimia.zarez11@gmail.com; 3 Medicinal Plants Research Center, Institute of Medicinal Plants, ACECR, Tehran, Iran; saeideh58_momtaz@yahoo.com; 4 Department of Toxicology and Pharmacology, School of Pharmacy, and Toxicology and Diseases Group, Pharmaceutical Sciences Research Center (PSRC), the Institute of Pharmaceutical Sciences (TIPS), Tehran University of Medical Sciences, Tehran, Iran; 5 GI Pharmacology Interest Group (GPIG), Universal Scientific Education and Research Network (USERN), Tehran, Iran; 6 Department of Clinical Science, Polytechnic University of Marche, Ancona, Italy; 7 Department of Life and Environmental Sciences, Polytechnic University of Marche, Ancona, Italy; t.bacchetti@staff.univpm.it; 8 Department of Academic Diabetes, Endocrinology and Metabolism, Hull York Medical School, University of Hull, Hull HU3 2JZ, UK; thozhukat.sathyapalan@hyms.ac.uk; 9 Department of Traditional Pharmacy, Mashhad University of Medical Sciences, Mashhad, Iran; emamia@mums.ac.ir; 10 Applied Biomedical Research Center, Mashhad University of Medical Sciences, Mashhad, Iran; 11 Biotechnology Research Center, Pharmaceutical Technology Institute, Mashhad University of Medical Sciences, Mashhad, Iran; 12 Department of Biotechnology, School of Pharmacy, Mashhad University of Medical Sciences, Mashhad, Iran

**Keywords:** Paraoxonase-1, Paraoxonase family, phytochemicals, diseases, inflammation, oxidative stress

## Abstract

Chronic diseases such as cardiovascular disease (CVD), atherosclerosis, chronic liver disease, and neurodegenerative diseases are major causes of mortality. These diseases have gained much attention due to their complications, and therefore novel approaches with fewer side effects are an important research topic. Free radicals and oxidative stress are involved in the molecular mechanisms of several diseases. Antioxidants can scavenge free radicals and mitigate their adverse effects. One of the most important antioxidant enzymes are paraoxonases (PONs). These enzymes perform a wide range of physiological activities ranging from drug metabolism to detoxification of neuroleptics. Paraoxonase-1 (PON1) is produced in the liver and then transferred to the bloodstream. It has been demonstrated that PON1 could have beneficial effects in numerous diseases such as atherosclerosis, CVD, diabetes mellitus, and neurodegenerative diseases by modulating relevant signalling pathways involved in inflammation and oxidative stress. These pathways include peroxisome proliferator-activated receptor gamma (PPAR-γ) and protein kinase B/nuclear factor kappa-light-chain-enhancer of activated B cells (AKT/NF-κB)-dependent signalling pathways. Increasing PON1 could potentially have protective effects and reduce the incidence of various diseases by modulating these signalling pathways. Several studies have reported that dietary factors are able to modulate PON1 expression and activity. This review aimed at summarizing the state of the art on the effects of dietary phytochemicals on PON1 enzyme activity and the relevant signalling pathways in different diseases.

## 1. Introduction

The incidence and prevalence of various diseases, specifically inflammatory disorders, are increasing in developed and developing countries. Cardiovascular disease (CVD), diabetes mellitus (DM), neurodegenerative disorders, atherosclerosis, and chronic liver injury are the substantial reasons for mortality and morbidity worldwide. It has been estimated that the prevalence of diseases correlated with lifestyle and eating habits, including DM and CVD, will increase in Asia and Middle East countries [1]. Different factors play essential roles in initiating these disorders, of which inflammation is the most crucial one. Increasing oxidative stress, inflammatory cytokines such as tumour necrosis factor-alpha (TNF-α), interleukin-1 (IL-1), interleukin-6 (IL-6), and other pro-inflammatory cytokines have pivotal roles in initiating and developing these common diseases. Hence, researchers are interested in investigating pathways in which oxidative stress and inflammatory cytokines play an integral role [2]. 

**Figure 1:**
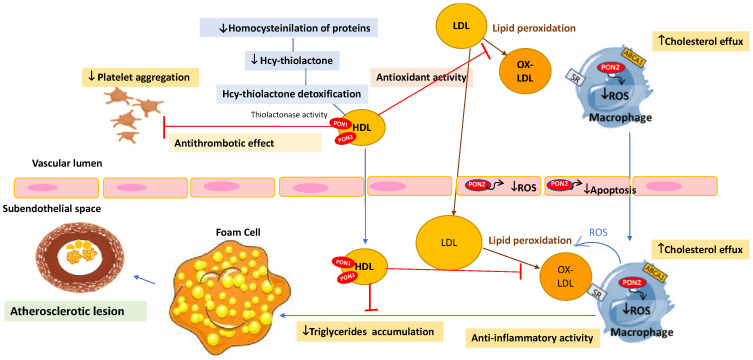
Pleiotropic antiatherogenic roles of PONs. Paraoxonase: PON; Paraoxonase 1: PON1; Paraoxonase 2: PON2; Paraoxonase 3: PON3; High-density lipoprotein: HDL; Oxidized low-density lipoprotein: Ox-LDL; Low-density lipoprotein: LDL; Reactive oxygen species: ROS.

Paraoxonase (PON) enzymes modulate inflammation and act as antioxidants in the human body. PON enzymes have been derived from the carboxylesterase enzymes. However, the gene and protein sequences of PON and carboxylesterases are not similar. Paraoxonase 1 (PON1), Paraoxonase 2 (PON2), and Paraoxonase 3 (PON3) have been recognised as PON family subgroups [3,4,5]. PON1 has been known as a hydrolyser or neutraliser of organophosphate xenobiotics such as paraoxon, soman, sarin, and other related toxic compounds. PON1 drew great attention after its discovery [6]. This enzyme is synthesized in the liver and generally circulated in the plasma associated with high-density lipoprotein (HDL). PON1 enzyme shows calcium ions sites and calcium is required for enzyme stability and catalytic activities [7]. PON1 can metabolise drugs that have lactone and cyclic carbonates in their chemical structures. In addition, PON1 can degrade homocysteine thiolactone and detoxify oxidised low-density lipoprotein (ox-LDL). The enzyme prevents LDL oxidation, the first step in the formation of foam-cell (Figure 1). These features make PON1 a protective factor in many disorders, especially atherosclerosis, cardiovascular, and related vascular diseases [8].

**Figure 2:**
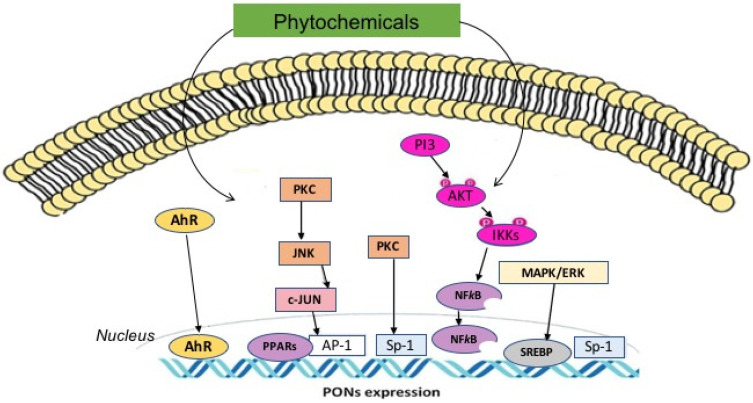
Effect of phytochemicals on signalling pathways that modulate PONs expression. AhR: aryl hydrocarbon receptor; PKC: Protein kinase C; JNK: c-Jun N-terminal kinase; PPARs: Peroxisome proliferator-activated receptors; AP-1: Activator protein 1; Sp-1Specificity protein-1; PI3K: Phosphoinositide 3-kinase; Akt: Protein kinase B; IKK: IK kinases; NF-κB: Nuclear factor-κB; MAPK/ERKs: mitogen-activated protein kinases/extracellular signal-regulated kinases; SREBPs: Sterol regulatory element-binding proteins.

Phytochemicals are extracted from herbal plants worldwide. These molecules have a variety of characteristics such as anti-inflammatory, antioxidant, and anti-ageing properties, which can potentially prevent various disorders [9,10,11,12,13,14]. Several studies have reported that phytochemicals could reduce pro-inflammatory cytokines such as tumour necrosis factor-alpha (TNF-α). They can increase the antioxidant ability via regulation of the PON1 enzyme [15]. Resveratrol, quercetin, curcumin, and compounds derived from plants including *Punica granatum*, *Cichorium intybus*, *Carthamus tinctorius* (Honghua)and *Salvia miltiorrhiza* (Danshen) could potentially prevent various diseases such as CVD, atherosclerosis, obesity, DM, hepatic disorders, and inflammatory diseases via affecting the PON1 signalling pathway [16]. The review summarizes the effect of phytochemicals on PON1 enzyme and relevant inflammatory signalling pathways (Table 1, Table 2 and Table 3) (Figure 2).

Paraoxonase (PON) is a group of enzymes encoded by three genes PON1, PON2, and PON3. They are located on chromosome 7 in a region that spans roughly 170 kb in humans. These genes are highly homologous and have a protein structure comparable to each other [54]. Each of those three genes has a higher comparative similarity in mammals, with 81–90% nucleotide sequence homology and 79–90% amino acids sequence homology [55]. PON2 appears to be the oldest PON gene based on structural homology and evolutionary distance [3]. PON2 is an intracellular enzyme found in a variety of tissues and organs. In contrast, PON1 and PON3 are produced by the liver and are secreted in blood associated to the high-density lipoprotein (HDL) [54]. 

PON1 is the most studied enzyme in the PON family and its role in cardiovascular disease (CVD), diabetes mellitus (DM), atherosclerosis, obesity, non-alcoholic fatty liver disease (NAFLD), and inflammatory diseases such as chronic liver inflammation has been reported [56].

PON1 is an HDL-associated lactonase that works in a Ca^2+^-dependent manner. HDL is required for PON1’s interaction with its substrates in addition to promoting its secretion from the liver [57]. PON1 is also generally contained in chylomicrons and very-low-density lipoproteins (VLDL), but to a lesser extent than HDL [58]. PON1 is not associated with LDL. PON1 was first discovered in mammalian tissues in the 1940s, which discovered an enzyme activity able to hydrolyse organophosphate insecticides [59]. In humans, the PON1 gene is found on chromosome 7 at 7q21–q22 (chromosome 6 in mice: the proximal region). The length of the human PON1 gene is about 26 kb. The coding structure contains nine exons, each with a splice donor and acceptor site like those seen in mammalian genes. In the presence of statins, the promoter part of the PON1 gene has connecting sites for sterol regulatory binding protein 2 (SREBP2), protein kinase C, and specificity protein 1 (Sp-1), all of which are thought to upregulate PON1. PPARs and the aryl hydrocarbon receptor have regulated the PON1 gene; however, binding sites have yet to be discovered [60]. 

PON1 is a glycoprotein with 355 amino acids in its structure and cysteine amino acids found in the 42, 284, and 353 positions. The aromatic structure of amino acids in the PON1 binding sites described the hydrophobicity of the PON1 active site (N-terminal portion) as a factor with organophosphatase function. PON1 is a glycoprotein and possesses four glycosylation positions in asparagine residues. 

PON1 has three different activities: arylesterase activity (which will be increased by apoA-1), lactonase activity, and organophosphate (paraoxonase) activity. Lipophilic lactones constitute the primary substrates of PON1. Hence, the lactonase activity of PON1 may have a significant role in health and diseases [61,62,63]. PON1 can also break down homocysteine-thiolactone, a reactive molecule able to induce protein N-homocysteinylation [64].

Various polymorphisms in the PON family lead to individuals’ different behaviors and genetic differences. More attention has been paid to the PON1 Q192R polymorphism. The two isoforms differ in their catalytic activity with many substrates. For example, the PON1 192R isoform hydrolyzes paraoxon (paraoxonase activity) faster than the 192Q isoform. Several studies demonstrate the association between Q192R and other polymorphisms, such as L55M, and susceptibility to coronary artery disease, but contrasting results have been reported [65,66].

PON1 is able to degrade lipid peroxides within the cell and lipoproteins in circulation, so the enzyme can inhibit LDL oxidation and prevent the formation of ox-LDL, which is the first step of atherosclerotic plaque generation. PON1 can suppress the transformation of monocytes to macrophages, which will prevent foam cells’ and atherosclerotic plaques’ formation (Figure 1). Furthermore, PON1 could reduce the secretion of monocyte Chemoattractant Protein-1 (MCP-1) by arterial endothelium in atherosclerosis. Finally PON1 can also increase the lysophosphatidylcholine (LPC) in macrophages, increase the HDL binding ability and promote the cholesterol efflux [67,68].

Apolipoprotein A-1 (apoA-1) and apolipoprotein J (ApoJ or clusterin), associated with HDL, were shown to enhance the stability of the PON1-HDL interaction [69,70,71]. Changes in serum PON1 activity and levels have been reported in several disorders involving oxidative stress, and the research on the role of PON1 in non-communicable disorders is increasing. 

Recent studies have reported that PON1 affects insulin sensitivity, glucose tolerance, and fasting blood glucose levels, and possesses functional ability in glucose metabolism regulation [72]. Using C57BL/6J and in PON1KO mice fed normal diet (ND) or high-fat diet (HFD) for 8 weeks, it has been demonstrated that PON1 deficiency caused enhanced insulin resistance in both ND and HFD mice. In fact, a decrease of glucose uptake in whole-body level, as reflected by a glucose tolerance test (GTT), by an insulin tolerance test (ITT) and by cellular glycogen accumulation in the liver and in the muscles, has been reported in PON1KO mice [28]. PON1 deficiency was also associated with increased oxidative stress, increased p38MAPK activity and attenuated insulin-mediated tyrosine phosphorylation of muscle insulin receptor substrate-1 (IRS-1), with a corresponding increase in serine phosphorylation [28]. 

In vitro studies have reported that PON1 addition to cultured C2 muscle upregulated glucose transporter 4 (GLUT4) expression. In fact, by inhibiting p38 mitogen-activated protein kinase (p38/MAPK) activity, PON1 can modulate GLUT4 expression and translocation, resulting in decreased insulin receptor substrate-1 serine phosphorylation and increased insulin receptor substrate-1 tyrosine phosphorylation [73]. PON1 disulfide groups and PON1 lactonase activity played a critical role in the modulation of the GLUT4 expression; in fact, the ability of PON1 to increase myocytes GLUT4 expression was partially inhibited upon blocking PON1 SH group, and completely abolished upon PON1 mutation in HIS115 of its catalytic site [73]. Nevertheless, using a metabolomic approach alterations of hepatic metabolism were observed in PON1-deficient fed a high-fat high-cholesterol diet and it was reported that PON1 has a detrimental effect on the levels of pentose phosphate pathway components such as xylonate, ribose 5-phosphate, and ribulose 5-phosphate, as well as fructose-1,6-biphosphate levels in the glycolysis [74]. Previous studies have reported a decrease of PON1 activity and of apoA-1 levels in type-2 diabetes (T2D) [75]. In inflammation, the activity and amount of PON1 will be decreased, and inversely, the expression of TNF-α and interleukin-1 (IL-1) would be increased [76].

## 2. Effect of Phytochemicals and Medicinal Plants on Paraoxonase-1 in Health and Disease

Several studies have investigated the effect of plant extracts or their phytochemicals on PON1 levels and activity. In vitro studies are summarized in Table 1. In vivo studies have also been carried out both in animal models (Table 2) and in human subjects (Table 3) in normal and pathological conditions. 

### 2.1. Moringa oleifera (Munga) and Its Bioactive Compounds (Beta-Sitosterol)

*Moringa oleifera* has been called “munga”, characterized by moringaceae and distributed in Himalayan tracts of Pakistan, Bangladesh, India, and Afghanistan. This horseradish tree is a fast-growing evergreen, known as a drum stick tree. Munga plants are rich in polyphenols such as quercetin, kaempferol and caffeoylquinic acid, and phytosterols such as beta-sitosterol [77]. In a recent study, Moradi et al. investigated the effects of three flavonoids, including kaempferol, galangin, and apigenin, from two different chemical subclasses of flavonoids on serum PON1 activity and stress oxidative parameters in male rats. They observed that the compounds increased the activity of PON1 in serum and inhibited MDA formation [27]. Munga phytochemicals exert their function by protecting liver mitochondria against oxidative damage. Munga leaf flavonoids can also preserve epithelial cells from oxidative stress induced by hydrogen peroxide in bovine mammary in vitro models [78,79]. Furthermore, munga leaf flavonoids promote vasorelaxation by blocking calcium channels and endothelium-dependent hyperpolarisation in mesenteric arterial beds isolated from the L-NAME hypertensive rat model [80]. Other studies reported the capacity of leaves extract of munga in improved testicular action, restored high fructose diet-induced via insulin resistance, and delayed the development of diabetes [81]. Sierra-Campos et al. have investigated the leaf extract of *Moringa oleifera* in a rat model with alloxan-induced diabetes [33]. Daily oral treatment with *Moringa oleifera* extract (200 mg/kg) increased the activity of endogenous antioxidants catalase (CAT) and PON1 (Table 2). Through molecular blind docking analysis, PON1 was found to have two binding sites for flavonoids that may be involved in the enzyme regulation [31]. Beta-sitosterol is one of the main bioactive compound in *Moringa oleifera* leave; it has chemoprotective and antidiabetic effects [82]. Moustafa et al. have investigated the beta-sitosterol effects on PON1 activity in rats exposed to gamma-radiation. Rats treated with beta-sitosterol show an increase in serum and hepatic PON1 activity, correlated with elevated expression of PPAR-γ (Table 2) (Figure 2). In addition, CAT, SOD, and high-density lipoprotein cholesterol (HDL-c) levels are increased. In contrast, cholesterol, triglyceride (TG), low-density lipoprotein cholesterol (LDL-c), and malondialdehyde (MDA) levels are reduced in serum of rats treated with beta-sitosterol [26] (Table 2). These results suggest that beta-sitosterol increasing endogenous antioxidants, including PON1, may be able to reduce the damaging effects of radiation and to improve the therapeutic index in radiation oncology treatments.

### 2.2. Euterpe oleracea Mart. (Açai)

*Euterpe oleracea Mart.* is initially distributed in the Amazon zone. Several studies reported that it contains polyphenols such as flavonoids involving proanthocyanidins (particularly cyanidin-3-rutinoside and cyanidin-3-glucoside) and anthocyanins, which exert antioxidant functions. In vivo and in vitro studies demonstrated that *Euterpe oleracea Mart* exerts a protective role against oxidative stress and promote anti-atherogenic, hypocholesterolemic, hepatoprotective, and anti-inflammatory effects [83]. Pereira et al. have explored the effect of filtered *Euterpe oleracea Mart.* pulp on PON1 expression and activity in a non-alcoholic fatty liver disease rat model [37]. Rats were fed with a high-fat diet (HFD), containing 1% cholesterol and 25% soy oil and treated with pulp of *Euterpe oleracea Mart.* (2 g/day) for six weeks. Treatment with *Euterpe oleracea Mart.* pulp reduced LDL oxidation, increased serum PON1 activity and its expression in the liver. Furthermore, *Euterpe oleracea Mart.* pulp reduced liver injury, decreasing TG and fat infiltration content [24] (Table 2).

### 2.3. Securigera securidaca (Adasolmolk or Gandeh Talkheh)

One of the Fabaceae family members is *Securigera securidaca,* commonly named “Adasolmolk” or “Gandeh Talkheh” in the traditional medicine of Iran, India, and Egypt since a long time ago. *Securigera securidaca* is considered an anti-hypertensive, antidiabetic, and anti-hyperlipidemia agent due to its multiple biological compounds such as flavonoids, tannins, phenolics, and saponins [84]. In an in vivo study, diabetic rats were treated with increasing doses of hydroalcoholic extract of *Securigera securidaca* seeds (100, 200, 400 mg/kg) alone and combined with glibenclamide (GB) (5 mg/kg) for 5 weeks. A higher dose (200, 400 mg/kg) of hydro-alcoholic extract of *Securigera securidaca* seeds alone or combined with GB improved lipid profiles and reduced cardiovascular disease risk indices. Moreover, the treatment reduced serum levels of markers of lipid peroxidation (MDA) and restored PON1 activity, while the levels of serum hs-CRP and TNF-α remained unchanged. Furthermore, a significant negative association between cardiovascular risk indices and the PON1 activity was reported [25] (Table 2).

### 2.4. Graptopetalum paraguayense

Graptopetalum concludes 12 species, involving *Graptopetalum paraguayense*, a folk herbal medicine famous as an anti-inflammatory and antioxidative herb in Taiwan and has been broadly administered for improving liver disease and renal disorders, CVD, and hypertension. Cheng et al. reported that 50% ethanol extract of *Graptopetalum paraguayense* increased PON1 gene expression by protein kinase B/nuclear factor kappa-light-chain-enhancer of activated B cells (AKT/NF-κB)-dependent signalling pathway in a human liver cancer cell line (HepG2 cells) model (Table 1) (Figure 2) [19].

### 2.5. Allium cepa L. (Onion) and Its Bioactive Compounds (Quercetin and Catechin)

*Allium cepa* or onion is one of the members of the Liliaceae family that is distributed worldwide and has been utilized as a food ingredient typically. *Allium cepa* contains several active components, including organosulfur compounds (propyl thiosulfinate), flavonoids (quercetin) that have antioxidant, anti-inflammatory, and anti-allergic properties [85]. Ulger et al. studied in vivo the alterations concerning the functional activity of onion induced by the heat treatment. Thirty-two rats were randomly assigned to receive four types of feed. Group I rat was fed a standard diet without induction of diabetes and group II rat received a standard diet with diabetes induction, group III was fed a diet supplemented with 5% lyophilized onion powder along with diabetes induction, and group IV used 5% oven-dried onion powder as diet supplements along with diabetes induction for 8 weeks. Diabetes was induced by streptozotocin injection (45 mg/kg). Total antioxidant status (TAS) and PON1 activity in group treated with lyophilized onion powder were higher than in other groups. These results indicate that onion treatment was able to increase PON1 activity and to reduce oxidative stress due to diabetes and that heat treatment adversely influences these onion properties [30] (Table 2). Jaiswal et al. explored the effect of onion extract and flavonoids (quercetin and catechin) in regulating PON1 expression and the levels of ox-LDL in rat model treated with mercuric chloride (HgCl_2_). Onion extract remarkably ameliorated HgCl_2_ side effects and reduced the levels of ox-LDL via upregulation of PON1 and its radical scavenging activity; the same effects were observed in rat treated with quercetin and to a lesser extent with catechin (Table 2) [40].

### 2.6. Ilex paraguariensis (Yerba Mate)

Mate tea is derived from dried leaves of *Ilex paraguariensis* and has been famous as a yerba mate. This beverage has a wide range of usage among South American countries, especially Uruguay, Argentina, Paraguay, and Brazil. Yerba mate concludes various classes of chemical compounds, including caffeoyl derivatives (caffeic acid, chlorogenic acid, 3,4-dicaffeoylquinic acid, 3,5-dicaffeoylquinic acid, and 4,5-dicaffeoylquinic acid); flavonoids (catechins, quercetin, kaempferol, and rutin); vitamin B2, B1, and C; amino acids; and minerals (phosphate, iron, and calcium) [86]. In vitro studies have demonstrated that *Ilex paraguariensis* extract (2–20 microL/mL) provided concentration- and time-dependent protection against AAPH-induced HDL oxidation, maintaining PON1 activity and apoA-1 structure [47] (Table 2). Moreover, an in vivo study demonstrated an increased plasma PON1 activity (about 10%) in healthy volunteers who received 0.5 L of *Ilex paraguariensis* extract compared to subjects who received 0.5 L of milk and coffee [49] (Table 3).

### 2.7. Punica granatum (Pomegranate) and Its Bioactive Compounds (Quercetin and Punicalagin)

*Punica granatum* is usually called pomegranate and is composed of various arils, seeds, and peel substances. In addition, pomegranate juice contains ellagitannin substances and punicalagin, which possess anti-atherogenic and antioxidant agents. Pomegranate and its phenolic compounds can activate PPAR-γ nuclear receptors, possibly regulating the promoter sequence of the PON1 gene. However, another member of the PON family, PON2, can be modulated by polyphenols of pomegranate by activation of PPAR-γ pathways (Figure 2) [87]. Khateeb et al. showed that polyphenols of pomegranate juice have a role in the upregulation of PON1 expression in the human hepatoma cell line (Table 1) [49]. Polyphenols of pomegranate juice increase PON1 expression through the intracellular signalling cascade PPAR/PKA/cAMP (Figure 2). The secreted PON1 displayed biological function and was able to protect HDL and LDL from in vitro induced oxidation [18]. In vivo studies carried out in a mice model fed a high-fat diet (HFD) demonstrated that pomegranate juice (PJ) administration decreased LDL-c, TC, TG, and increased HDL-c. The risk ratio and atherogenic index were remarkably improved in HFD-mice treated with PJ. Higher PON1 activity was reported in HDF-mice treated with PJ and its activity was correlated to HDL levels. PJ treatment was also associated with a decrease of serum levels of markers of lipid peroxidation (TBARS) and an increase of serum GSH levels [50]. Moreover, in the same experimental model, PJ treatment induced PON2 upregulation and a decrease in macrophage [22] (Table 2). The effect of quercetin and punicalagin, the main polyphenols in pomegranate, on glucose levels, PON1 activity, and inflammatory responses were investigated in the mice model by Atrahimovich et al. [23]. Balb/c mice were fed with a high-fat diet (HFD) for 12 weeks; in the last month, mice received subcutaneous treatments through implanted minipumps able to release daily in serum physiological levels of atorvastatin, quercetin, and punicalagin. The HFD decreased serum PON1 lactonase activity, whereas treatment with punicalagin restored PON1 activity to the average level in mice with a regular diet. Moreover, punicalagin restored HDL functions, as demonstrated by the increased anti-inflammatory properties in HDL isolated from HFD-mice treated with punicalagin. Punicalagin administration reduced serum glucose concentrations in HFD- mice [23] (Table 2).

### 2.8. Red Wine and Grape Seed Extract (GSE)

Red wine has been known as an enriched source of polyphenol compounds. Many studies have assessed the effect of moderate intake of red wine on the PON1 enzyme [88]. Navarro-García et al. investigated the effect on PON1 activity of dietary intake, red wine consumption, and three genotypes of PON1 (Q192R, L55M, and C-108T) in healthy people. Forty-five healthy Mexican volunteers ingested 120 mL of red wine per day for 6 weeks. Neither PON1 genotypes nor dietary intake impacted PON1 activity. Nevertheless, an increase in AREase activity of PON1 was observed in participants after wine consumption [47] (Table 3). Kiyici et al. reported that grape seed extract (GSE) administration was able to increase PON1 activities in streptozotocin-induced diabetic rats [39] (Table 2). Noll et al. investigated the effect of a red wine polyphenolic extract consumption on PON1 in a murine model with hyperhomocysteinemia induced by deficiency of cystathionine β-synthase (CBS). Polyphenolic extract of red wine was added for 4 weeks to the drinking water of CBS-deficient mice, fed with a high-methionine diet. The supplementation with a low dose of red wine polyphenolic extract decreased homocysteine levels and increased hepatic and plasma PON1 activity [41] (Table 2).

### 2.9. Berberis vulgaris (Barberry)

Barberry (*Berberis vulgaris*) is a red-coloured fruit distributed in Europe and Asia. This fruit includes antioxidant compounds such as berberine, berberrubine and berbamine. Multiple pharmacological properties have been considered in the traditional medicine of Iran and Turkey for barberry involving antipyretic, antibacterial, antiarrhythmic, and antipruritic. In addition, the barberry root has therapeutic actions, such as stimulating the immune system, reducing fever, and restoring appetite. Recent findings reported that berberine, an ingredient of barberry extract, improved glucose metabolism by glycolysis stimulation and lowered insulin resistance. Barberry juice significantly improved diabetes complications and lipid profile [51,89]. Forty-two patients were included in the clinical study of Lazavi et al. and received barberry juice and a placebo for 8 weeks. At the end of the study, the systolic blood pressure, diastolic blood pressure, total cholesterol (TC), fasting blood sugar, and triglycerides (TG) were significantly reduced in the group treated with barberry juice. In addition, plasma PON1 levels increased in the barberry juice received group compared with the control group [51] (Table 3).

### 2.10. Flos Carthamus tinctorius L. (Honghua) and Radix Salvia miltiorrhiza (Danshen)

Clinical studies have demonstrated that a standardized water-soluble complex containing *Flos Carthamus tinctorius* L. (Honghua) and *Radix Salvia miltiorrhiza* (Danshen) can attenuate the clinical symptoms in patients suffering from coronary heart disease (CHD). However, its exact mechanism remains elusive. Sue et al. reported that SOD and PON1 activity in old CHD patients was drastically lower than in old patients without CHD in a clinical experiment. Treatment for 4 weeks, with this standardized water-soluble complex containing *Flos Carthamus tinctorius* L. (Honghua) and *Radix Salvia miltiorrhiza* (Danshen), restored the PON1, SOD, and MDA level [90] (Table 3).

### 2.11. Fragaria ananassa (Strawberries)

*Fragaria ananassa* or strawberries are enriched with polyphenols, ellagic acid and ellagitannins, and anthocyanins, anti-inflammatory, and antioxidant activities. Some anthocyanins such as pelargonidin-*O*-glucuronide were determined in human plasma post of the meal, including strawberries. A diet supplemented with strawberries decreases TC and LDL in serum and reduces peroxidative damage to LDL in obese persons, including those with metabolic syndrome [91]. In addition, obese patients with hyperlipidemia who consume strawberries inhibited the postprandial elevation of ox-LDL and TG in reply to HFD [92]. Moreover, strawberries can decrease pro-oxidant processes, causing oxidative stress in healthy subjects by increasing plasma antioxidants and reducing the production of reactive oxygen species through reposing phagocytes in peripheral blood [93]. Strawberry polyphenols may elevate the gene transcription of nuclear factor-erythroid 2 related factor 2 (Nrf2) that regulates the biosynthesis of different antioxidant enzymes [94]. Furthermore, they can have an impact on activity and expression of PPARs involved in several cellular processes such as lipid metabolism and fatty acid oxidation [95]. Zasowska-Nowak et al. investigated the effect of strawberry administration on the activity of plasma PON1 and lipid profile in healthy subjects. Thirty-one patients (BMI: 24.5 ± 4.0 kg/m^2^) with the usual diet received 500 g/day strawberry pulp for 1 month, and after 10 days’ washout, the cycle was repeated once. The results demonstrated that supplementation of strawberries with a regular diet reduced PON1 activity and did not improve lipids profiles in healthy subjects without obesity [48] (Table 3).

### 2.12. Rhus coriaria (Sumac)

“Sumac” is a common name of *Rhus coriaria* L. attributed to the Anacardiaceae family. Sumac is broadly distributed in Southern Italy, Spain, Turkey, Middle Eastern countries, Afghanistan, and Iran. Sumac is traditionally consumed as a spice in food. Anti-inflammatory, antimicrobial, antimutagenic, antimalarial, antithrombin, antitumorigenic, antioxidant, antiviral, cytotoxic, leukopenic, and hypoglycemic effects have been reported [96]. The effect of sumac on PON1 was investigated in a double-blind, randomized placebo-controlled trial on 41 T2D volunteers. Participants were randomly divided into two groups and the treated group received 3 g/day sumac powder, and the placebo group received an equivalent concentration of placebo for 3 months. The results demonstrated a significant increase of plasma PON1 activity and a decrease of plasma levels of insulin, hs-CRP, MDA in the sumac-received group [50] (Table 3).

### 2.13. Vaccinium macrocarpon (Red Cranberry)

*Vaccinium macrocarpon* or red cranberry fruits consist of various phytochemicals with different effective properties in human biological actions. The advantages of cranberry in preventing urinary tract infection have drawn considerable attention. Recently, scientists found that cranberry phytochemicals can attenuate risk factors and prevent some types of cancers, CVD, infectious diseases, and neurological disorders [97]. Nevertheless, there are few clinical documents to provide these findings. Cranberry compounds inhibit or interact with drugs, although cranberry products are assumed to be safe and non-toxic for humans. Clinical trials have demonstrated that using large amounts of cranberry juice does not change the warfarin pharmacodynamics and its anticoagulant activity [98]. In a double-blind, randomized clinical trial, 58 T2D male patients were randomly assigned to consume 1 cup/day cranberry juice or a placebo drink for 12 weeks. Fasting blood sugar was assessed at the beginning and the 12th week of the study. There was a considerable reduction in serum apoB, glucose, and a significant increase in serum apoA-1 and PON1 activity in the cranberry juice group compared to the control group [53] (Table 3).

### 2.14. Phoenix dactylifera (Date Palm)

*Phoenix dactylifera* L., commonly called “date palm”, is distributed in northern Africa and South-West Asia. The study of phytochemical composition of date fruit reported that it contains phenolics, sterols, carotenoids, anthocyanins, flavonoids, and procyanidins that are free radical scavenger, antimutagenic, hepatoprotective, anti-inflammatory, antioxidant, and nephroprotective factors [99]. Takaeidi et al. investigated the effects of methanolic date seed extract (DSE) on AREase and PON activities in the hypercholesterolemic rat models. Rats were divided into two groups (regular and hypercholesterolemic groups) for 4 weeks. Two weeks after using the regular and hypercholesterolemic diet, various DSE dosages were received during the last 2 weeks of the treatment. DSE administration remarkably increased AREase and PON serum activities in treated groups compared to untreated groups. In addition, there was a high difference in the serum TAS between the hypercholesterolemic and regular diet groups [28] (Table 2).

### 2.15. Canola Oil

Plant-derived lipids, especially canola oil and olive oil, are rich in monounsaturated fatty acids (MUFA), and plant stanols and sterols. Stanols’ and sterols’ consumption reduce LDL and TC plasma levels in animals and humans, through inhibition of intestinal cholesterol absorption [100]. Fuhrman et al. investigated the effect of canola oil (CO) and a formulation of soybean sterols esterified to fatty acids from CO (PS-CO) in Apo E ^−/−^ Mice. CO and PS-CO administration decreased plasma TC, TG, and lipid peroxide levels compared to placebo. PON1 activity was significantly reduced after CO consumption in comparison to placebo, while PS-CO treatment did not affect the enzyme’s activity. Furthermore, peritoneal macrophages from PS-CO-fed mice showed decreased ox-LDL cellular uptake compared with placebo-fed mice [32] (Table 2).

### 2.16. Resveratrol (3,5,4’-trihydroxystilbene)

Resveratrol has been known as a polyphenolic stilbene compound (3,5,4’-trihydroxystilbene) and is considered a phytoalexin that is distributed in various plants such as blueberries, plums, peanuts, apples, grapes, and oilseeds [101,102]. Resveratrol has several biological activities. Antiplatelet, anti-inflammatory, and antioxidant properties are some of the beneficial biological effects of resveratrol [103], though some studies have questioned the beneficial effects of this phytochemical in clinical practice [104,105,106]. In addition, resveratrol has shown its capacity to suppress lipid peroxidation and to reduce LDL and TG levels in vivo. Resveratrol can modulate gene expression, and it has been reported to antagonise transcription of genes, including sensitive transcription factors to oxidative stress such as Activator protein 1 (AP-1) or NF-κB [101]. Gouedard et al. demonstrated that resveratrol increased PON1 gene expression in the hepatoma cell line and human hepatocyte cultures (Table 1). The effect of resveratrol in promoting the PON1 gene was independent on estrogen receptors and was facilitated via aryl hydrocarbon receptor (AhR) and an unusual AhR responsive factor [17] (Figure 2). Chen et al. have demonstrated that an intravitreal injection of resveratrol restored the retinal PON1 expression and vascular permeability in streptozotocin-induced diabetic rats. Moreover, STZ-diabetic rats treated with resveratrol for 12 weeks showed a significant reduction of plasma glucose and insulin levels and an increase of plasma PON1 activity. Treatment with resveratrol induced an increase of PON1 expression and a decrease of expression of retinal inflammatory factors (IL-1β, IL-6, TNF-α, VEGF, IFN-γ and MCP-1) and caspase activation in retina of STZ-diabetic rats [73] (Table 2). Additionally, an in vitro study demonstrated that resveratrol decreased the caspase3 expression and activity in high glucose-stimulated rat retinal endothelial cells. Nevertheless, PON1 silencing increased the damage that was induced by HG and reduced the protection of resveratrol rat retinal endothelial cells [29] (Table 1). In the double-blinded randomised trial of Tabatabaei et al., 71 patients with T2D received a placebo (methylcellulose) or 1000 mg/day resveratrol capsules for 8 weeks. They reported that resveratrol increased PON1 activity in comparison with placebo in patients with T2D [52] (Table 3).

### 2.17. Quercetin (3,3’,4’,5,7-pentahydroxyflavone)

One of the significant flavonols in several vegetables and fruits, such as berries, onions, capers, and apples, is quercetin (3,3’,4’,5,7-pentahydroxyflavone). Daily intake of all flavonoids has been considered 200–300 mg while using flavonols is approximately 20 mg/day (quercetin accounts for nearly 50%), with a daily intake of quercetin is about 10 mg/day but it can reach more than 200 mg/day. As mentioned above, the highest concentration of quercetin has been detected in *Allium cepa* L. (onion), *Asparagus officinalis* L. (Asparagus), and *Lactuca sativa* L. (red leaf lettuce), with lower levels in tomatoes, peas, broccoli, and green peppers. In addition, apples are among the most valuable fruit source for quercetin levels, as well as cherries and different types of berries [107]. Garige et al. demonstrated that quercetin modulated PON1 gene expression via sterol regulatory element binding protein 2 that translocates from the endoplasmic reticulum to the nucleus, where it specifically interacts with a sterol responsive element–like sequence in paraoxonase 1 promoter in HuH7 liver cells [21] (Table 1). Ibrahim et al. reported the quercetin treatment was able to increase PON1 hepatic gene expression, to enhance the cellular antioxidant system and to exert a protective effect against hepatic damage induced in rats following gestational exposure to fenitrothion [43] (Table 2). Boesch-Saadatmandi et al. explored hepatic PON1 status in response to apolipoprotein E (apoE) genotype and dietary intake of quercetin supplementation in a mice model. ApoE3 and apoE4 transgenic mice were fed for 6 weeks a semi-synthetic diet without quercetin or 2 mg/g quercetin. PON1 hepatic mRNA and protein levels were substantially higher in apoE3 than apoE4 mice. Feeding quercetin-enriched diets promoted gene expression of hepatic PON1, and the effect was higher in apoE3 compared to apoE4 mice [44] (Table 2).

Furthermore, Leckey et al. reported that quercetin and moderate ethanol suppressed atherosclerosis development via increasing PON1 hepatic expression, with a concomitant increase of serum PON1 activity in a mice model [45] (Table 2). Gong et al. demonstrated that administration of quercetin (10 mg/L) in the liquid diet for 4 weeks significantly increased hepatic PON1 expression and PON1 serum activities, including serum PON1 homocysteine thiolactonase activity, compared to the control group. HDL from quercetin group had higher protective properties in in vitro induced LDL oxidation compared to HDL from the control group [46] (Table 2). An increase of PON1 and ARE activities was observed in a rat model of oxidative stress associated with renal failure induced by ethylene glycol (EG) after treatment with quercetin [90] (Table 2).

### 2.18. Curcumin

Curcumin is a polyphenolic compound of turmeric that is commonly known for its effective pharmacological activities [108,109,110,111,112,113,114] involving hepatoprotective, antifibrotic, and anti-inflammatory activities due to the respective capacities for scavenging the free radicals [115,116]. Recently, many studies have been designed to investigate the effect of curcumin on the PON1 enzyme activity [117]. Schrader et al. assessed the impact of curcumin on PON1 transactivation in cultured hepatocytes. However, the molecular and cellular actions are not completely clear. Schrader et al. investigated the effect of dietary curcumin (500 mg/kg) for 2 weeks in mice and no significant effect of curcumin on the expression of PON1 was reported [20] (Table 2). Khodarahmi et al. evaluated the effect of curcumin (100 mg/kg/day) on PON1 gene expression and activity in rat undergone bile duct ligation, a model of chronic liver disease associated with oxidative damage [83]. Results revealed that treatment with curcumin reduced changes in hepatic enzymes, fibrotic markers mRNA expression, and liver histology. Moreover, curcumin upregulated PON1 gene expression and increased the expression of regulatory genes attributed to the PON1 gene expression such as Protein kinase C-α (PKC-α), SREBP2, specificity Protein 1 (Sp1), c-Jun N-terminal kinase (JNK), and AhR (Figure 2). Moreover, an increase of PON1 activity associated with Apo-A1 upregulation has been reported [33] (Table 2). Altintoprak et al. investigated the curcumin antioxidant effects in an allergic rhinitis rat model. Curcumin treatment for 4 weeks increased PON1 and SOD serum activity and tissue GSH values [34] (Table 2). Treatment with curcumin increased PON1 mRNA and led to a high increase in serum PON1 and homocysteine thiolactonase activities in an ethanol-induced hepatic injury rat model [35] (Table 2). Yildirim et al. investigated the effect of curcumin on PON1 activities in dextran sulphate sodium (DSS)-induced ulcerative colitis mice. The outcomes demonstrated that prophylactic administration of curcumin restored serum and liver PON activities, which were reduced by DSS [36] (Table 2). Fatolahi et al. examined the effect of exercise rehabilitation and curcumin administration on rats’ hepatocyte injuries induced by forced drinking ethanol [87]. Their findings revealed that the interaction of swimming training and curcumin induces an increase PON1 gene expression and a reduction of NF-κB gene expression [37] (Table 2). Roxo et al. investigated the effect of a yogurt enriched with metformin and curcumin, alone or as mixtures on PON1, glycoxidative stress, and metabolic parameters in diabetic rats. Treatment with metformin or curcumin individually reduced the glucose, TG, cholesterol, and TBARS plasma levels and increased plasma PON1 activity in diabetic rats. Moreover, curcumin combined with metformin showed a synergy effect on dyslipidemia and TBARS levels [38] (Table 2).

## 3. Conclusions and Future Perspectives

Free radical high exposure and insufficient response of antioxidants in the human body have been involved in the molecular mechanisms of several disorders such as atherosclerosis, liver diseases, CVD, DM, and inflammatory diseases. Different factors, such as dietary phytochemicals, could improve the antioxidant function in humans. Different phytochemicals such as curcumin, resveratrol, quercetin, and plant extracts, including *Ilex paraguariensis*, *Punica granatum*, and *Berberis vulgaris,* can modulate antioxidant capacity and inflammation through multiple signalling pathways, particularly PON1 enzymes and downstream signalling pathways, due to their broad range of physiological activities such as drug metabolism and detoxification of lipid peroxidation products. Several in vitro and in vivo studies investigated the capacity of phytochemicals to modulate PON enzymes, especially PON1 activity in normal and pathological conditions. The results reported that the several phytochemicals and extracts increased PON1 activity as well as PON1 gene expression, which lead to decreased levels of inflammatory cytokines such as TNF-α, IL-1β, IL-6 and oxidative stress. More studies are required to elucidate the role of PON1 in CVD, atherosclerosis, DM, and related symptoms. Furthermore, novel investigations should be required to assess the bioavailability, metabolism, and the effectiveness of phytochemicals. Generally, multiple clinical studies must be also designed to evaluate the potential of phytochemicals as PON1 regulators in different phases of diseases, particularly atherosclerosis, CVD, and DM. 

## Figures and Tables

**Table 1:** Effects of phytochemicals on PON1 signalling pathways: in vitro studies.

Cell Type	Phytochemical Treatment	Intervention		Treatment Duration	Results	Ref.
		Case	Control			
Human hepatoma cell line (HuH7)	Resveratrol (RSV)	RSV (10 µmol/L)	Ethanol 0.1%	48 h	↑ PON1 gene promoter activity through aryl hydrocarbon receptor (AhR) and an unconventional AhR responsive element ↑ PON1 mRNA levels	[17]
Human hepatoma cell line (HuH7)	Pomegranate juice (PJ)/ Punicalagin/ Gallic acid/ or Ellagic acid	0.36 mmol/L	untreated cell	24 h	↑ PON1 expression and activity via PPAR-γ/PKA/cAMP pathway ↓ levels of TBARS in LDL and HDL oxidized by copper ions	[18]
HepG2 cells	Graptopetalum paraguayense (GP)	GP in water (30, 100, 300 µg/mL) GP in ethanol 50% (30, 100, 300 µg/mL) GP in ethanol 95% (30, 100, 300 µg/mL)	untreated cell	48 h	↑ enzymatic activities of secreted PON1 ↑ IKKα/β ↓ IKBα ↑ PON1 mRNA expression via Akt/NF-kB pathway (GP in ethanol 50% with 100 and 300 µg/mL concentration)	[19]
Human hepatoma cell line (HuH7)	Curcumin	Curcumin (1, 5, 10, 15, 20 µmol/L)	RSV (25 µmol/L)	48 h	↑ PON1 transactivation (10 µmol/L and higher concentration of curcumin)	[20]
Human hepatoma cell line (HuH7)	Quercetin	10, 20 µmol/L	DMSO 0.1%	48 h	↑ PON1 level and activity ↑ PON gene transcription via binding SREBP2 to SRE-like sequence in the PON1 promoter	[21]

Abbreviations: Resveratrol: RSV; Cardiovascular disease: CVD; aryl hydrocarbon receptor: AhR; increase/upregulate: ↑; Pomegranate juice: PJ; Protein kinase A: PKA; Cyclic adenosine monophosphate: cAMP; Peroxisome proliferator-activated receptor-gamma: PPAR-γ; Low-density lipoproteins: LDL; High-density lipoprotein: HDL; Human liver cancer cell line: HepG2 cells; Protein kinase B: Akt; Nuclear factor kappa-light-chain-enhancer of activated B cells: NF-κB; Inhibitory κB kinases: IKK; Inhibitory κB: IκB; Paraoxonase 1: PON1; Dimethyl sulfoxide: DMSO; Sterol regulatory binding protein 2: SREBP2; Graptopetalum paraguayense: GP; Pomegranate juice: PJ.

**Table 2:** Effects of phytochemicals on PON1: in vivo studies.

Mouse Strain	Treatment	Disease	Intervention		Number of Animals		Treatment Duration	Results	Ref.
			Case	Control	Case	Control			
CD1 Mice	Punica granatum (Pomegranate)	HFD-induced obesity	HFD; HFD + APJ (200 µL, o.g.); HFD + EPJ (200 µL, o.g.); HFD + APJ (200 µL, o.g.) + EPJ (200 µL, o.g.);	Normal diet	*n* = 5/group		6 month	↑ AREactivity ↓ serum TG,TC,LDL-c, and atherogenic index ↑ HDL-c ↑ GSH levels ↓ serum TBARS levels ↑ mouse peritoneal macrophages (MPM)PON2 activity ↓ MPM mediated oxidation of LDL ↓ MPM uptake of ox-LDL	[22]
Balb/c Mice	Quercetin	HFD-induced obesity	HFD + vehicle ((DDW + 2% Tween 80), s.c. implanted minipump); HFD + punicalagin (140 µg/100 µL, s.c. implanted minipump); HFD + quercetin (42 µg/100 µL, s.c. implanted minipump); HFD + atorvastatin (15 mg/100 µL, s.c. implanted minipump);	Normal diet	*n* = 18/group		12 weeks	Punicalagin: ↑ PON1 lactonase activity ↑ HDL anti-inflammatory activity evaluated by dichlorofluorescein cell-free assay ↓ glucose levels	[23]
Female Rats Fischer 344	*Euterpe oleracea* Mart (Açai) (EO)	Non-alcoholic fatty liver disease (NAFLD)	HFD; EO (2 g/day/single dose, o.g.); HFD + EO (2 g/day/single dose, o.g.);	AIN-93M (control diet)	*n* = 8/group		6 weeks	↑ PON1 and apoA-1 expression in the liver ↑ serum/hepatic PON1 activity ↓ serum ox-LDL levels ↓ hepatic injury markers (ALT) ↓ hepatic TG levels	[24]
Male Wistar Rats	Hydroalcoholic extract of *Securigera securidaca* seeds (HESS)	STZ-induced Diabetes mellitus (DM)	HESS (100, 200, 400 mg/kg, p.o.); HESS (100, 200, 400 mg/kg, p.o.) + Glibenclamide (Gb) (5 mg/kg, p.o.); Gb (5 mg/kg, p.o.).	No treatment	*n* = 6/group		5 weeks	200 and 400 mg/kg HESS alone and in combination with GB: ↓ cardiovascular risk lipid indices ↑ serum PON1 activity ↓ serum MDA levels	[25]
Male Wistar Rats	Beta-sitosterol (BS)	Gamma irradiation-induced oxidative stress	BS (40 mg/kg/day, oral); Irradiation + Saline (0.5 mL/day, oral) Irradiation + BS (40 mg/kg/day, oral);	Saline (0.5 mL/day, oral)	*n* = 6/group		10 days	↑ serum and hepatic PON1 activity ↓ TG, TC, and LDL-c ↑ HDL-c ↓ MDA ↑ PPAR-γ gene expression ↑ SOD, CAT activity	[26]
Female B6C3F1 Mice	Curcumin	-	Normal diet + Curcumin (500 mg/kg, p.o.)	Normal diet	*n* = 8/group		2 weeks	no effect	[20]
Male Wistar Rats	Kaempferol/Galangin/Apigenin	-	Kaempferol (10 mg/kg/day, p.o.); Kaempferol (20 mg/kg/day, p.o.); Galangin (10 mg/kg/day, p.o.); Galangin (20 mg/kg/day, p.o.); Apigenin (10 mg/kg/day, p.o.); Apigenin (20 mg/kg/day, p.o.); Vehicle (Ethanol 10%, p.o.)	Normal diet	*n* = 5/group		2 months	↑ serum PON1 activity ↓ MDA production Kaempferol > Galangin > Apigenin	[27]
Male NMRI Rats	Methanolic date seed extract (DSE)	Hypercholesterolemia (HC)	HC + DSE (250 mg/kg/day, p.o.); HC + DSE (500 mg/kg/day, p.o.); HC + DSE (1000 mg/kg/day, p.o.); HC; DSE (1000 mg/kg/day, p.o.); HC + atorvastatin (10/ mg/day, p.o.); Atorvastatin (10/ mg/day, p.o.);	Normal diet	not mentioned		4 weeks	↑ serum PON1 and ARE activities	[28]
Male Sprague-Dawley (SD) Rats	Resveratrol(RSV)	STZ-induced DM	RSV (0.1 and 1 µg/mL/day, intravitreal);	Vehicle (PBS, 5 µL, intravitreal);	*n* = 5/group		24 h post-injection	↑ PON1 mRNA levels in retina ↑ VEGF and bFGF mRNA levels in retina ↓ blood LDL and ↑ HDL (1 µg/mL/day)	[29]
			RSV (5, 10,50 µg/kg/day, TVI)	Vehicle (PBS, equal volume, TVI);			12 weeks	RSV 10 μg/kg and 50 μg/kg/day: ↑ plasma PON1 activity ↓ blod glucose and insulin levels ↑ retinal PON1 mRNA levels ↓ retinal AGEs levels and apotosis ↓ retinal caspase3 activation ↓ plasma ox-LDL ↓ retinal inflammatory factors (IL-1β, IL-6, TNF-α, VEGF, IFN-γ and MCP-1)	
Male Wistar Rats	*Allium cepa*	STZ-induced DM	DM + Normal diet; DM + diet including 5% onion powder (dried at −76 °C in lyophilizator); DM + diet including 5% onion powder (dried at +80 °C in furnace);	Normal diet	*n* = 8/group		8 weeks	Lyophilized onion powder: ↑ PON1 activity ↑ Total Antioxidant Capacity ↓ Total Oxidant Status	[30]
Male Wistar Rats	*Moringa oleifera* leaves extract(MOLE)	Alloxan-induced DM	DM; DM + MOLE (200 mg/kg/day, o.g.);	Distilled water	*n* = 5/group		3 weeks	↑ serum PON1 lactonase activity ↑ serum CAT activity	[31]
Apo E ^−/−^ Mice	Canola oil	Atherosclerosis	PS-CO, soybean sterols esterified to fatty acids from Canola oil (2.5 mg/day, oral); CO, Canola oil;	PBS	*n* = 5/group		10 weeks	*PS-CO treatment*: No effect on PON1 activity ↓ TC and TG ↑ TAS ↓ Ox-LDL retention from MPM	[32]
Male Wistar Rats	Curcumin	Chronic liver disease induced by bile duct ligation (BDL)	BDL + Curcumin (100 mg/kg/day, o.g.); Curcumin (100 mg/kg/day, o.g.); BDL (vehicle (CMC), o.g.);	No treatment	*n* = 8/group		4 weeks	↑ PON1 activity ↑ PON1 expression ↑ expression of Sp-1, JNK, AhR, SREBP2, PKC-α	[33]
Female Wistar Rats	Curcumin	Allergic rhinitis (AR)	AR + azelastine HCl (intranasal, from day 21 to 28, twice a day); AR + Curcumin (200 mg/mL, 20 µL/nostril, intranasal, from day 21 to 28, twice a day)	No treatment	*n* = 8–10/group		4 weeks	↑ serum PON1 activity ↑ serum SOD activity ↑ tissue GSH level, ↑ Serum GSH-Px activity ↓ Tissue MDA levels	[34]
Female Wistar-Furth rats	Curcumin	Ethanol-induced hepatosteatosis	LFO + ethanol (35% of dietary calories derived from ethanol); HFO + ethanol (35% of dietary calories derived from ethanol); HFO + ethanol (35% of dietary calories derived from ethanol) + Curcumin (150 mg/kg/day, oral); LFO + ethanol (35% of dietary calories derived from ethanol) + Curcumin (150 mg/kg/day, oral);	LFO; HFO;	*n* = 4/group		8 weeks	↑ PON1 mRNA ↑ serum PON1 activity ↑ PON1 homocysteine thiolactonase activity	[35]
Female Balb/c Mice	Curcumin	DSS-induced ulcerative colitis	Ulcerative colitis (5% DSS + water, o.g.); Ulcerative colitis + Sulfasalazine (dissolved in olive oil, o.g.); Ulcerative colitis + Curcumin (dissolved in olive oil, o.g.);	(Water + olive oil, o.g.)	*n* = 7/group		1 week	↑ serum PON1 activity ↑ MPO activity ↓ Weight loss ↑ Colon lengths	[36]
Male Wistar Rats	Curcumin	Ethanol-induced hepatotoxicity	Ethanol-curcumin (50 mg/kg, i.p., 5 times per week); Ethanol- swimming training (5 times per week); Ethanol- swimming training (5 times per week) + curcumin (50 mg/kg, i.p., 5 times per week);	Dextrose-control; Ethanol-control; Ethanol-saline; Ethanol- DMSO;	*n* = 8/group		2 weeks	↑ PON1 gene expression ↓ NF-κB gene expression	[37]
Male Wistar Rats	Curcumin	STZ-induced DM	DM treated with yoghurt; DM treated with yoghurt + Curcumin (90 mg/kg/day); DM treated with yoghurt + Metformin (250 mg/kg/day); DM treated with 4 U/day insulin	Normal rat treated with yoghurt	*n* = 10/group		1 month	↑ plasma PON1 activity ↓ Plasma levels of glucose ↓ TG, TC, TBARS ↓ fluorescent advanced glycation end products (AGEs)	[38]
Male SD Rats	Grape Seed Extract (GSE)	STZ-induced DM	GSE (100 mg/kg/day); DM; DM + GSE (100 mg/kg/day);	No treatment	*n* = 6–10/group		6 weeks	↑ serum PON1 activity	[39]
Male SD Rats	*Allium cepa* Quercetin Catechin	HgCl_2_-induced oxidative stress	Onion extract (10 mL/kg/day, o.g.); Quercetin (20 mg/kg/day, o.g.); Catechin (20 mg/kg/day, o.g.); HgCl_2_ (5 mg/kg, i.p.); Onion extract (10 mL/kg/day, o.g., 10 days before HgCl_2_ (5 mg/kg, i.p.)); Catechin (20 mg/kg/day, o.g., 10 days before HgCl_2_ (5 mg/kg, i.p.)); Quercetin (20 mg/kg/day, o.g., 10 days before HgCl_2_ (5 mg/kg, i.p.));	No treatment	*n* = 6/group		4 weeks	↑ PON1 activity ↑ plasma radical scavenging activity ↓ plasma ox-LDL ↓ plasma MDA	[40]
CBS ^+/−^ Mice	Red wine polyphenolic extract	Hyperhomocysteinemia	Methionine diet; Methionine diet + Low polyphenolic extract (LPE) (which contains 25 μg of catechin, and 12 mg of polyphenols); Methionine diet + High polyphenolic extract (HPE) (which contains 100 μg of catechin	No treatment	*n* = 6/group		4 weeks	↑ Plasma and hepatic PON1 activity ↑ PON1 expression ↑ CBS activity ↓ plasma MDA ↓ Plasma homocysteine ↓ ox-LDL	[41]
Male Wistar Rats	Quercetin Catechin Epicatechin	Ethylene glycol-induced renal failure (EG)	EG (water, oral); EG + Quercetin (100 mg/L, 5.5 mg/kg, oral); EG + Catechin (100 mg/L, 5.5 mg/kg, oral); EG + Epicatechin (100 mg/L, 5.5 mg/kg, oral); EG + a folk herbal extract *Fagolitos* (7 ml/L, 0.4 ml/kg, oral);	No treatment	*n* = 9/group		16 days	↑ PON1 and ARE activities↑ Citrate synthase and SOD activity ↑ PON1/apoA-1 ratio ↓ oxidative damages (SOD and Citrate synthase activities were not modified by EG treatment but SOD activity was increased by Catechin, and Citrate synthase activity was increased by Quercetin, Catechin and folk herbal extract Fagolitos)	[42]
Pregnant Rats	Quercetin	Organophosphorus-induced hepatic apoptosis	Quercetin (100 mg/kg, o.g.); Fenitrothion (4.62 mg/kg, o.g.); Quercetin (100 mg/kg, o.g., 2 h before taking Fenitrothion) + Fenitrothion (4.62 mg/kg, o.g.);	Distilled water	*n* = 10/group		2 weeks	↑ PON1 hepatic gene expression ↑ TAS ↑ SOD and CAT activity ↑ GSH level	[43]
Apo E ^−/−^ Mice	Quercetin	Atherosclerosis	Quercetin-enriched diets (2 mg per g diet)	Normal diet	*n* = 8/group		6 weeks	↑ PON1 expression and activity via PPAR-γ pathway	[44]
LDLR ^−/−^ Mice	Quercetin	Atherosclerosis	Atherogenic diet + 18% ethanol calories; Atherogenic diet + 25% ethanol calories; Atherogenic diet + quercetin 12.5 mg/dL; Atherogenic diet + quercetin 18.75 mg/dL; Atherogenic diet + quercetin 25 mg/dL;	Atherogenic diet	*n* = 6/group		8 weeks	↑ PON1 hepatic gene expression ↑ PON1 activity ↓ decreases in aortic lesions	[45]
Male Wistar Rats	Quercetin	LDL oxidation	Quercetin-enriched liquid diets (10 mg/L);	Normal diet	*n* = 6/group		4 weeks	↑ PON1 hepatic gene expression ↑ PON1 activity ↑ serum PON1 homocysteine thiolactonase ↓ LDL oxidation	[46]

Abbreviations: Coronary heart disease: CHD; High diet fat: HFD; Resveratrol: RSV; Cardiovascular disease: CVD; Protein kinase C-α: PKC-α; c-Jun N-terminal kinase: JNK; Aryl hydrocarbon receptor: AhR; increase/upregulate: ↑ and decrease/downregulate: ↓; Pomegranate juice: PJ; Protein kinase A: PKA; Cyclic adenosine monophosphate: cAMP; Peroxisome proliferator-activated receptor-gamma: PPAR-γ; Low-density lipoproteins: LDL; High-density lipoprotein: HDL; Human liver cancer cell line: HepG2 cells; Protein kinase B: Akt; Nuclear factor kappa-light-chain-enhancer of activated B cells: NF-κB; Inhibitory κB kinases: IKK; Inhibitory κB: IκB; Paraoxonase 1: PON1; Dimethyl sulfoxide: DMSO; Sterol regulatory binding protein 2: SREBP2; Pomegranate juice: PJ; Saudian pomegranate juice: APJ; Egyptian pomegranate juice: EPJ; Oral gavage: o.g.; Triglyceride: TG; Total cholesterol: TC; High-density lipoprotein cholesterol: HDL-c; Low-density lipoprotein cholesterol: LDL-c; Glutathione: GSH; Double distilled water: DDW; Subcotaneus: s.c.; Non-alcoholic fatty liver disease: NAFLD; *Euterpe oleracea* Mart: EO; oral: p.o.; Apolipoprotein A-1: apoA-1; Diabetes mellitus: DM; Hydroalcoholic extract of *Securigera securidaca* seeds: HESS; Streptozotocin: STZ; Glibenclamide: Gb; Tumor necrosis factor-alpha: TNF-α; high-sensitivity C-reactive protein: hs-CRP; Beta-sitosterol: BS; Malondialdehyde: MDA; Arylesterase: AREase; Methanolic date seed extract: DSE; Hypercholesterolemia: HC; Vascular endothelial growth factor: VEGF; Interferon-gamma: IFN-γ; Monocyte chemoattractant protein-1: MCP-1; Sprague-Dawley: SD; Tail vein injection: TVI; Oxidized low-density lipoprotein: ox-LDL; Total Antioxidant Status: TAS; *Moringa oleifera* leaves extract: MOLE; Apolipoprotein E: apoE; Phosphate-buffered saline: PBS; Bile duct ligation: BDL; Carboxymethyl cellulose: CMC; Allergic rhinitis: AR; Glutathione peroxidase: GSH-Px; Low ω-3 PUFA: LFO; High ω-3 PUFA: HFO; Dextran sulphate sodium: DSS; Intraperitoneally: i.p.; Intravenous: i.v.; Thiobarbituric acid reactive substances: TBARS; Grape seed extract: GSE; Mercuric chloride: HgCl_2_; cystathionine β-synthase: CBS; Low polyphenolic extract: LPE; High polyphenolic extract: HPE; Ethylene glycol-induced renal failure: EG; LDL receptor: LDLR; Superoxide dismutase: SOD.

**Table 3:** Effects of phytochemicals on PON1: clinical studies.

Study Design	Phytochemical Type	Disease	Intervention		Number of Patients		Treatment Duration	Results	Ref
			Case	Control	Case	Control			
Clinical randomized trial	Red wine (tannin, gallotannin)	-	Red wine (120 mL/day, contain alcohol 12.5 %)	-	45	-	6 weeks	↑ PON1 and ARE activities	[47]
Clinical randomized trial	*Fragaria ananassa* (Strawberry)	-	Usual diet + strawberry (500 g/day)	Usual diet	31	20	1 month	↓ PON1 activity = TC, TG, LDL levels	[48]
Clinical randomized trial	*Ilex paraguariensis*(IP)	-	500 mL IP	500 mL milk, coffee, or nothing	2	2	1 day	↑ PON1 activity	[49]
Double-blind, randomized placebo-controlled	*Rhus coriaria* (Sumac)	Type 2 diabetes (T2D)	Sumac (3 g/day, oral)	Placebo (3 g/day, oral)	22	19	3 months	↑ PON1 activity ↓ MDA ↓ hs-CRP ↓ Insulin	[50]
Clinical randomized trial	*Berberis vulgaris* (Barberry)	T2D	*Berberis vulgaris* juice (200 mL/day)	No treatment	23	23	8 weeks	↓ Systolic blood pressure ↑ PON1 activity ↓ Fasting blood glucose ↓ TG	[51]
Double-blind, randomized placebo-controlled	Resveratrol	T2D	Resveratrol capsule (1000 mg/day)	Placebo capsule (methylcellulose, 1000 mg/day)	35	36	8 weeks	↑ PON1 activity ↓ Asymmetric de-methyl-arginine	[52]
Double-blind, randomized placebo-controlled	*Vaccinium macrocarpon* (Cranberry)	T2D	Cranberry juice (240 mL/day)	Natural mineral water with strawberry flavor (240 mL/day)	29	29	12 weeks	↑ PON1 activity ↑ PON1 activity ↑ apoA-1 levels ↓ apoB levels ↓ glucose levels	[53]

Abbreviation: Arylesterase: AREase; Paraoxonase 1: PON1; Malondialdehyde: MDA; Low-density lipoproteins: LDL; Triglyceride: TG; Total cholesterol: TC; *Ilex paraguariensis*: IP; C-reactive protein: hs-CRP; Type 2 diabetes: T2D; Apolipoprotein B: apoB; Apolipoprotein A-1: apoA-1.2. Paraoxonase-1 in health and diseases. Increase/upregulate: ↑; decrease/downregulate: ↓

## Abbreviations

Paraoxonase: PON; High-density lipoprotein: HDL; Oxidized low-density lipoprotein: ox-LDL; Sterol regulatory binding protein 2: SREBP2; Specificity protein 1: Sp-1; Peroxisome proliferator-activated receptors: PPARs; Low-density lipoproteins: LDL; Very low-density lipoproteins: VLDL; Apolipoprotein J: ApoJ; Chylomicron: CM; Apo lipoprotein: APO; Cardiovascular disease: CVD; Diabetes mellitus: DM; Non-alcoholic fatty liver disease: NAFLD; Lysophosphatidylcholine: LPC; Common antioxidant enzyme: CAT; Interleukin-1: IL-1; Tumor necrosis factor: TNF; 4: GLUT 4; PON1 disulphide groups: SH; L-N^G^-Nitro arginine methyl ester: L-NAME; Catalase: CAT; Paraoxonase 1: PON1; Paraoxonase 2: PON2; Paraoxonase 3: PON3; Peroxisome proliferator-activated receptor gamma: PPAR-γ; Superoxide dismutase: SOD; High-density lipoprotein cholesterol: HDL-c; Low-density lipoprotein cholesterol: LDL-c; High-fat diet: HFD; Triglyceride: TG; Protein kinase C-α: PKC-α; c-Jun N-terminal kinase: JNK; Aryl hydrocarbon receptor: AhR; Glutathione: GSH; Dextran sulphate sodium: DSS; Protein kinase B: Akt; Nuclear factor kappa-light-chain-enhancer of activated B cells: NF-κB; Thiobarbituric acid reactive substances: TBARS; Malondialdehyde: MDA; Tumor necrosis factor-alpha: TNF-α; high-sensitivity C-reactive protein: hs-CRP; Protein kinase A: PKA; Cyclic adenosine monophosphate: cAMP; Total cholesterol: TC; Arylesterase: AREase; Grape seed extract: GSE; Cystathionine β-synthase: CBS; Activator protein 1: AP-1; Interleukin: IL; Vascular endothelial growth factor: VEGF; Interferon-gamma: IFN-γ; Monocyte chemoattractant protein-1: MCP-1; Type 2 diabetes: T2D; Monounsaturated fatty acids: MUFA; Carbon tetrachloride: CCl_4_; Glutathione peroxidase: GPx; Coronary heart disease: CHD; Nuclear factor-erythroid 2 related factor 2: Nrf2; Body mass index: BMI; Apolipoprotein B: apoB; Apolipoprotein A-1: apoA-1; Total Antioxidant Status: TAS; Mercuric chloride: HgCl_2_; Date seed extract: DSE; Apolipoprotein E: apoE; Human liver cancer cell line: HepG2 cells; Interleukin 1 beta: IL-1β; Glucose transporter 4: GLUT4; p38 mitogen-activated protein kinase: p38/MAPK.
